# Granulomatous mastitis in a male breast: A case report and review of literature

**DOI:** 10.1002/ccr3.7048

**Published:** 2023-03-02

**Authors:** Kei Kawashima, Shinya Yamamoto, Kazutaka Narui, Yoshie Fujiwara, Shoko Adachi, Mahato Sasamoto, Masanori Oshi, Akimitsu Yamada, Eita Kumagai, Masako Otani, Itaru Endo

**Affiliations:** ^1^ Department of Breast and Thyroid Surgery Yokohama City University Medical Center Yokohama Japan; ^2^ Department of Gastroenterological Surgery Yokohama City University Graduate School of Medicine Yokohama Japan; ^3^ Department of Pathology Yokohama City University Medical Center Yokohama Japan

**Keywords:** granulomatous mastitis, lymphoma, male breast, neoplasms

## Abstract

Granulomatous mastitis (GM) is a rare disease, particularly among men. Herein, we present a case of GM diagnosed in a 63‐year‐old male patient who showed reduction in the tumor size during 3 months of observation.

## Graphical abstract

The optimal treatment strategy for granulomatous mastitis in men is still controversial due to its rarity. However, observation may be a viable option in asymptomatic cases.

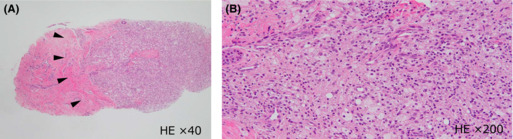

## 1 INTRODUCTION

Granulomatous mastitis (GM), a chronic inflammatory disorder of the breast with unknown etiology, was first described by Kessler et al. in 1972.^1^ GM usually affects parous females and is rarely diagnosed in male breasts. We describe a rare case of GM diagnosed in a male patient during the therapeutic course of follicular lymphoma.

## 2 CASE HISTORY

**FIGURE 1:**
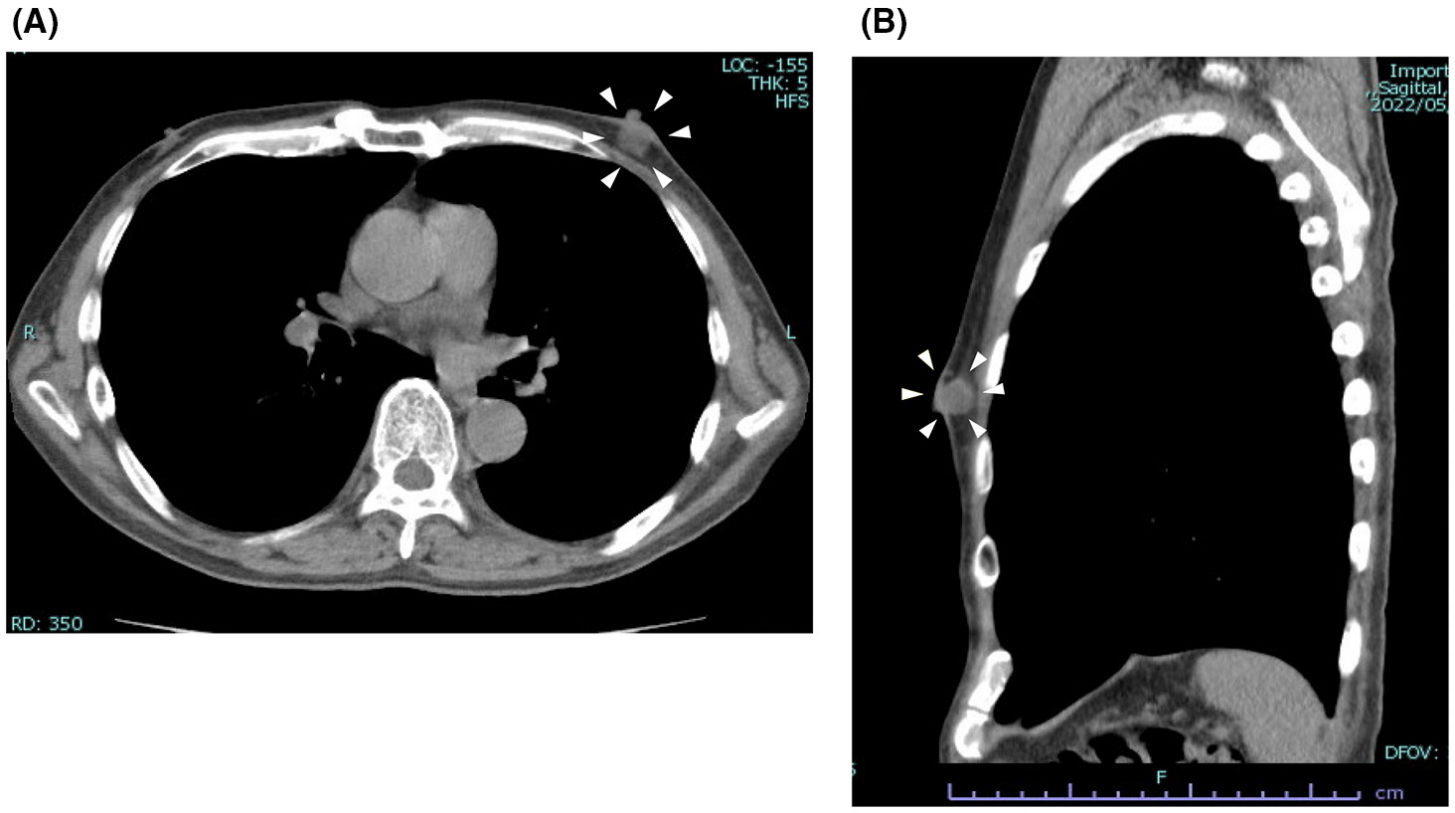
Plain computed tomography scan (A) coronal plane, (B) axial plane at the first visit. A tumor measuring 13.9 × 12.9 × 6.1 mm in the subareolar portion of the left breast (Arrowhead).

**FIGURE 2:**
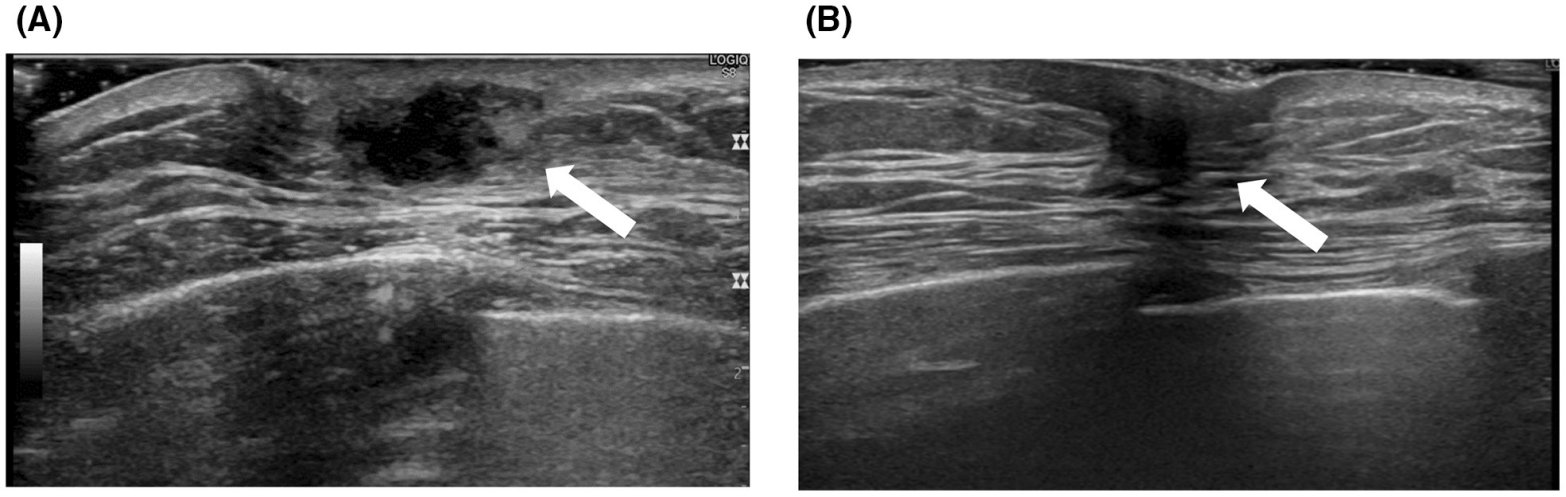
(A) Ultrasonogram at the first visit. An ill‐defined hypoechoic irregular mass with peripheral vascularity (Arrow). (B) Ultrasonogram after 3 months. Tumor size reduced to 7.9 × 5.5 × 5.2 mm (Arrow).

**FIGURE 3:**
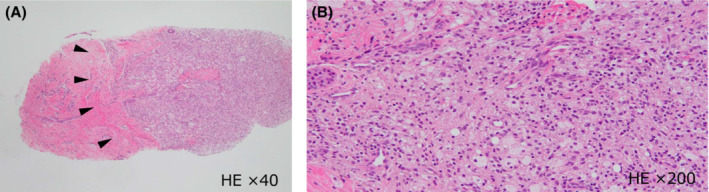
Histopathological findings of the core needle biopsy specimen (A) HE ×40 magnification, (B) HE ×200 magnification. Infiltration of mononuclear inflammatory cells accompanied by foam cells (Arrowhead). HE, hematoxylin and eosin stain.

A 63‐year‐old male patient with history of chemotherapy for follicular lymphoma presented with a left breast mass detected on a periodic computed tomography (CT) scan. He was diagnosed with follicular lymphoma 5 years ago and treated with six cycles of a combination of rituximab and bendamustine, followed by treatment with rituximab for 2 years as maintenance therapy. Recurrence of lymphoma was detected at the cervical and axillary lymph nodes after 2 years of an uneventful post‐therapeutic course. Lenalidomide and rituximab were administered as treatment for recurrence. A CT scan performed at the end of chemotherapy showed disappearance of all target lesions, but appearance of a new mass in his left breast. The patient was referred to our department for further evaluation. On physical examination, a mass measuring approximately 1 cm in diameter was palpable in the subareolar area of his left breast, while no swollen lymph nodes were detected in the ipsilateral axillary region. Laboratory tests including tumor marker analyses were all within normal levels (white blood cell, 2840/μL; C‐reactive protein, 0.032 mg/dL; carcinoembryonic antigen, 3.8 ng/mL; cancer antigen 15–3, 17 U/mL; sIL‐2R, 385 U/mL). CT scan confirmed the presence of a lesion measuring 13.9 × 12.9 × 6.1 mm in the subareolar portion of the left breast (Figure 1). Breast ultrasonography revealed an ill‐defined hypoechoic irregular mass with peripheral vascularity in the subareolar portion (Figure 2A). Ultrasound‐guided core needle biopsy was performed, and histological examination showed a granulomatous structure comprising mononuclear inflammatory cell infiltration accompanied by foam cells without any evidence of caseous necrosis (Figure 3). No specific pathogen or foreign bodies were detected. These findings confirmed the diagnosis of GM.

Since there was no suspicion of malignancy and the patient was asymptomatic, he was admitted for observation. Ultrasonography performed after 3 months revealed that the size of the tumor was 7.9 × 5.5 × 5.2 mm (Figure 2B), which was smaller compared with the initial diagnosis. The patient is currently under observation.

## 3 DISCUSSION

GM is diagnosed histologically by confirming the presence of non‐necrotizing granuloma formation, without any evidence of microorganisms such as Mycobacterium or fungi.^2^ Its etiology is uncertain, and imaging findings are not specific. GM occurs in female breasts and accounts for 1.8% of benign breast disorders proven by biopsy.^3^ It mainly affects females of reproductive age. The mean age at presentation is 33–38 years.^1^, ^4^, ^5^ Hormonal disruption and autoimmune responses have been suggested as the possible etiological factors.^6^

GM is rarely observed in males. To the best of our knowledge, there are only 16 reported cases of GM in male patients.^1^, ^2^, ^4^, ^5^, ^6^, ^7^, ^8^, ^9^, ^10^, ^11^, ^12^, ^13^, ^14^, ^15^ The reported cases have been summarized in Table 1. We analyzed 12 cases of patients whose characteristics and clinical courses were described.^2^, ^6^, ^7^, ^8^, ^9^, ^10^, ^11^, ^12^, ^13^, ^14^, ^15^ The median age of the patients at the time of presentation was 46 years (range: 17–60 years). Eight patients (73%) had GM in the right breast, while two (17%) presented with bilateral involvement. The median size of the masses was 20 mm (range: 5–72 mm). All patients had a breast mass with or without pain, while two (17%) had ulcerations. Nipple discharge was observed in only one patient (8%). A definitive diagnosis of GM was obtained by core needle biopsy in eight patients (67%), by fine needle aspiration in one patient (8%), and by excisional biopsy in three patients (25%). The baseline characteristics of female GM patients have been described as follows: mean age of 36 years, lesion measuring approximately 50 mm, and nipple discharge observed in 30% patients.^16^

**TABLE 1:** Reported cases of granulomatous mastitis in male patients.

S. No	Author/Year	Country	Age	Associated clinical features	Breast affected	Size (mm)	Diagnostic technique	Treatment	Outcome	Follow‐up period (months)	Comorbidities
1	Heer et al.^7^ 2003	United Kingdom	60	None	Left	20	CNB	N/A	N/A	N/A	None
2	Reddy et al.^8^ 2005	United Kingdom	46	None	Right	35	FNA	Local excision	N/A	N/A	None
3	Al Manasra et al.^9^ 2016	Jordan	29	None	Right	13	US	Excisional biopsy	No recurrence	3	None
4	Moris et al.^6^ 2017	Greece	53	None	Right	63	CT	Excisional biopsy	No recurrence	18	None
5	Sam et al.^2^ 2017	United States	36	None	Right	5	CNB	Observation	N/A	N/A	Estrogen therapy (transgender patient)
6	Joo et al.^10^ 2018	Korea	60	None	Right	12	CNB	Corticosteroids	Regression	3	Gynecomastia
7	Sahin et al.^13^ 2019	Turkey	49	None	Right	13	CNB	Observation	Regression	6	None
8	Farrokh et al.^11^ 2019	Iran	46	None	Right	24	CNB	Corticosteroids	Regression	6	None
9	Dinc et al.^12^ 2020	Turkey	58	Nipple retraction	Right	13	CNB	Corticosteroids	No recurrence	6	None
10	Yin et al.^14^ 2020	China	17	Ulcer	Bilateral	72	MRI	Excisional biopsy with drainage	N/A	N/A	Pituitary tumor Gynecomastia
11	Maszima et al.^15^ 2022	Canada	46	Nipple retraction	Bilateral	34	CNB	Corticosteroids + MTX	No recurrence	24	Gynecomastia
12	Yin et al.^16^ 2022	China	20	Nipple discharge	Left	26	CNB	Bromocriptine	Regression	3	Gynecomastia
13	Korkut et al.^5^ 2015	Turkey	N/A	–	–	–	–	–	–	–	–
14	Prasad et al.^1^ 2017	India	N/A	–	–	–	–	–	–	–	–
15	Sarica et al.^17^ 2018	Turkey	N/A	–	–	–	–	–	–	–	–
16	Barreto et al.^4^ 2018	United States	21	–	–	–	–	–	–	–	–
17	Our case 2022	Japan	63	None	Left	14	CNB	Observation	Regression	3	Lymphoma

Abbreviations: CNB, core needle biopsy; FNA, fine needle aspiration; MTX, methotrexate; N/A, not applicable.

One possible reason for rarity in males is the absence of mammary lobules, which are usually affected during this disease. It has been reported that estrogen stimulation causes the development of acini and lobules in male breasts^2^ and might be responsible for the development of GM. Of the 12 cases, four patients had gynecomastia^10^, ^14^, ^15^, ^16^ and one was a transgender (male to female transition, receiving estrogen therapy for 6 years).^2^ This supports the fact that GM in males might be associated with abnormal hormonal conditions such as gynecomastia and estrogen therapy. This may also be responsible for the higher age of affected male patients compared with female patients, since the ratio of androgen to estrogen in males lowers with age.^18^ However, hormonal involvement was not detected in the remaining seven cases and our case. Therefore, it can be concluded that the etiology of GM in males remains unclear.

The optimal treatment strategy for GM remains controversial. Treatment approaches include observation, oral corticosteroid administration, and surgical excision. Incision and drainage may be a treatment option for bacterial infection cases. Oral corticosteroids have been used as the first‐line treatment in some studies and reported to be effective in decreasing the size of GM.^4^ However, observation without any therapeutic intervention could be considered for asymptomatic cases. Some studies have reported that GM patients who underwent observation without any medication achieved resolution in 7–14.5 months.^19^, ^20^

Of the 11 reported male cases in which treatment was mentioned, four patients underwent surgical excision. Core needle biopsy was not attempted in these four cases, and the lesion was surgically excised in toto as an excisional biopsy. Of the remaining seven patients, four who received oral corticosteroid therapy were symptomatic, whereas two who underwent observation did not have any symptoms other than the breast mass. In one patient who was treated with bromocriptine for pituitary tumor, the breast tumor disappeared after the treatment. None of the patients experienced recurrence during their clinical course, but the follow‐up periods were not sufficient to determine the long‐term outcomes. In our case, since the patient was asymptomatic and diagnosis of GM was confirmed histologically, we decided to observe the lesion.

In contrast to other reported cases, the tumor in our case appeared during the clinical course of follicular lymphoma treatment. Although not histologically proven, one possible explanation for the occurrence of GM in our case may be the autoimmune response toward the extranodal lesion of lymphoma in the breast.

To summarize, we presented a rare case of GM in a male patient. Observation may be a viable option in the asymptomatic cases. However, further studies including a larger number of patients and longer observation period are needed for reliable long‐term outcomes.

## AUTHOR CONTRIBUTIONS

**Shinya Yamamoto:** Supervision. **Kazutaka Narui:** Supervision. **Yoshie Fujiwara:** Supervision. **Shoko Adachi:** Supervision. **Mahato Sasamoto:** Supervision. **Masanori Oshi:** Supervision. **Akimitsu Yamada:** Supervision. **Eita Kumagai:** Supervision. **Masako Otani:** Supervision. **Itaru Endo:** Supervision.

## CONFLICT OF INTEREST STATEMENT

The authors declare no conflicts of interest regarding the publication of this article.

## ETHICAL APPROVAL

This study was conducted ethically in accordance with the World Medical Association Declaration of Helsinki.

## CONSENT

Written informed consent was obtained from the patient to publish this report in accordance with the journal's patient consent policy.

## Data Availability

Data sharing is not applicable to this article as no datasets were generated or analyzed during the current study.
